# Intravenous immunoglobulin therapy: usage patterns and response to treatment in Qatar over ten years

**DOI:** 10.3389/fimmu.2024.1481079

**Published:** 2024-12-02

**Authors:** Salma A. Taha, Sherin Thalappil, Ramzy M. Ali, Haajra Fatima, Asaad Omer A. Imameldin, Sami Aqel, Ahmed M. Abdelaal, Timo Siepmann, Jessica Barlinn, Maryam A. Al-Nesf

**Affiliations:** ^1^ Allergy and Immunology Division, Department of Medicine, Hamad Medical Corporation, Doha, Qatar; ^2^ General Internal Medicine Division, Department of Medical Education, Hamad Medical Corporation, Doha, Qatar; ^3^ Department of Pharmacy, Hamad Medical Corporation, Doha, Qatar; ^4^ Division of Health Care Sciences, Dresden International University, Dresden, Germany; ^5^ Department of Neurology, University Hospital Carl Gustav Carus, Technische Universität Dresden, Dresden, Germany

**Keywords:** human immunoglobulin, intravenous immunoglobulin (IVIg), IVIg clinical indications, IVIg adverse reactions, IVIg therapy outcome, United States Food and Drug Administration (FDA), European Medicines Agency (EMA)

## Abstract

**Background:**

IVIg is a blood-derived antibody product initially designed as a replacement therapy in inborn errors of immunity (IEIs). However, over the last 50 years, IVIg has been used to treat a growing range of autoimmune, autoinflammatory, and secondary immunodeficiency disorders. The US FDA has licensed IVIg for use in the treatment of nine clinical indications; although, IVIg global usage extends to off-label indications with variable treatment responses. Data from Qatar on the use of IVIg is scarce; thus, hampering the formulation of local policies. This study aimed to examine the utilization patterns, clinical indications, and safety profile of IVIg usage in Qatar; a nation with a predominantly young population, and to investigate the response rates to short- and long-term IVIg treatment, as well as explore associations between age at first IVIg dose, clinical indication, and treatment response.

**Methods:**

A retrospective chart review was conducted of patients who received IVIg between March 2009, and March 2019, in Hamad General Hospital, Qatar. Demographics, immediate adverse effects of IVIg, and treatment response were collected. IVIg clinical indications were categorized into FDA- and/or EMA-approved, those supported by international guidelines; those approved as second-line therapy, and those with low or no supportive evidence.

**Results:**

IVIg was used for 63 indications during the 10-years. The age of patients skewed towards a younger demographic (median (IQR) 24 (44-6) years); however, no significant differences in response to short- and long-term treatment between age groups were observed. Of the 841 patients, 62% received IVIg in concordance with international recommendations, while 14% bestowed the treatment for indications with low or no supportive evidence. Immediate IVIg adverse effects were documented in 4% of patients in all of the infusions received, with headaches being the most prevalent (1.8%). Variable treatment responses were observed, with the highest recovery reported in immune thrombocytopenic purpura (35%), followed by transverse myelitis (28%).

**Conclusion:**

This study provided crucial insights into IVIg utilization, safety, and treatment outcomes in Qatar’s young population. Despite variability in treatment responses and off-label use, adherence to international recommendations remained eminent. Further research is warranted to inform local guidelines and optimize IVIg therapy outcomes.

## 1 Introduction

Human immunoglobulin (Ig) is a pivotal plasma-derived medicinal product in clinical medicine. It is available in many forms, such as subcutaneous, intramuscular or intravenous (IVIg), but the latter is the most commonly used. More than 15 different IVIg brands are available worldwide with variable immunoglobulin-G content.

IVIg has been used for multiple indications since its introduction in 1980. It was primarily approved for immunodeficiencies for the prevention and treatment of recurrent infections. Since then, there has been an increased utilization of IVIg as an immunomodulatory agent for a diverse list of autoimmune and inflammatory disorders. The clinical specialties using the largest amounts of IVIg are neurology, hematology, immunology, nephrology, rheumatology, and dermatology (1).

Currently, there are only a few approved indications for the use of IVIg, as established by the United States Food and Drug Administration (FDA) and the European Medicines Agency (EMA) (2, 3). These indications include IEI, primary immune thrombocytopenic purpura (ITP), Kawasaki disease (KD), B-cell chronic lymphocytic leukemia (CLL), chronic inflammatory demyelinating polyradiculoneuropathy (CIDP), multifocal motor neuropathy (MMN), dermatomyositis (DM), post bone marrow transplantation and in children with HIV infection. Nevertheless, IVIg has shown growing prominence in several off-label indications, and a recent review of off-label usage of IVIg in neurological conditions demonstrated that, while pediatric Guillain-Barré syndrome (GBS), and myasthenia gravis have shown promising results with IVIg treatment, success with other conditions like epilepsy and acute disseminated encephalomyelitis lacks supportive evidence (4).

Few studies have examined predictors of positive outcomes in IVIg therapy. Among them, a study of patients with GBS found that older age negatively impacted disease outcomes (5). Similarly, a study on KD showed that older children, male sex and laboratory abnormalities were risk factors for IVIg non-response (6). In both adult and pediatric groups treated with first- and second-line ITP therapy, age ≥ 6 years and platelets ≥ 20 x 10^9/^L were identified as risk factors for chronic ITP (7). Disease heterogeneity, lack of a unified definition of a favorable outcome, as well as the complexity of the outcome measuring tools were some of the challenges faced when attempting to accurately predict IVIg treatment outcomes.

The loss of tolerance and the presence of class-switched autoreactive IgG antibodies are central features in autoimmune and autoinflammatory diseases. Yet, infusing IVIg at high doses into patients with ITP and CIDP, among others, has exhibited curative effects. The major component of IVIg is the purified IgG, which has a wide range of specificities since it is derived from a large, diverse pool of healthy donors. At low doses, IVIg delivers passive immunity capable of opsonizing and neutralizing common bacterial and viral pathogens essential for immunodeficiency patients. It can also activate cellular and innate immunity. On the contrary, IVIg administered at doses of 1-3 g/kg has immunoregulatory effects, primarily through the complex interaction between the IgG neonatal fragment crystallizable receptor (FcRn) and the Fcγ receptor family (FcγRs) on immune and non-immune cells (8).

The surge in IVIg consumption over the past decade has led to worldwide shortages, which prompted many countries to develop and refine guidelines and shortage plans to control the dispensing process of IVIg, and to ensure it is available for patients most in need. The high cost of IVIg is a major limitation. A 3-year study conducted in the Kingdom of Saudi Arabia showed that 43.65 kg of IVIg was consumed during their study period with an estimated cost of 1.75 million USD, of which 24.4% was used for off-label indications (9).

Nearly three million people are currently residing in the Qatar peninsula. The population is uniquely young (median age 32 years), with a mixture of indigenous and expatriate groups.

Plasma fractionation services are not yet established in Qatar to cover the local demand, and IVIg is mostly imported. The average international cost of IVIg can vary; however, a US study (10), in 2014 reported an annual cost of almost 10,000 USD for a single infusion of immunoglobulin; thus, making IVIg therapy one of the most expensive treatments, particularly when used for chronic diseases. Furthermore, there are no national regulations in Qatar governing IVIg usage to ensure safe and appropriate use of this costly medication.

We conducted a comprehensive 10-year retrospective analysis of IVIg utilization patterns in Hamad General Hospital (HGH), which is the main tertiary healthcare facility in Qatar. We aimed to assess thoroughly the spectrum of both approved and off-label indications and evaluate treatment response and safety profile for IVIg, leveraging real-world data spanning a decade. Additionally, we explored the relationship between age at first dose of IVIg, clinical indications and response to treatment for any significant interactions. This in-depth analysis will provide valuable insights for shaping regulatory decisions regarding the appropriate use of IVIg internationally and locally within Qatar’s healthcare system.

## 2 Materials and methods

### 2.1 Design, setting and study population

A retrospective, observational study was conducted using routinely collected health data to evaluate the administration and safety of intravenous immunoglobulin (IVIg) products in Hamad General Hospital (HGH) in the ten-year time period from 2009-2019.

HGH is a 600-bed tertiary care hospital serving more than one million residents in the city of Doha and surrounding districts. It provides multiple health services, including specialized medical and surgical services, general pediatrics, emergency medicine and a trauma center. Solid organ transplant services started in 2009 with renal transplant, followed by liver transplant in 2011. In 2015, HGH transitioned to an electronic information system using the Cerner *millennium* ^®^ platform health record. Before January 2018, HGH was the main provider of pediatric services in Qatar, offering specialized pediatric units for tertiary medical care.

In HGH, all prescribing clinicians are authorized to request IVIg, and no special committee approval is required. If the pharmacy team raises a clarification regarding the indication of IVIg, two senior physicians must co-sign the prescription for approval. The hospital’s drug supply chain and the main pharmacy are responsible for purchasing and distributing IVIg, which is provided free of charge to patients as it is considered a life-saving medication.

All HGH pharmacy records of IVIg prescriptions between March 25, 2009, and March 24, 2019, were retrieved and linked to their respective patients in the electronic health record (EHR). For prescriptions earlier than 2015, scanned copies of paper files and the older electronic health record (Medicom^®^) were also examined. Any patient with one or more completed prescription orders of intravenous immunoglobulin was included in the study. Prescriptions not linked to patients and canceled/voided prescriptions were excluded. A total of 20 patients received subcutaneous immunoglobulin and their data was previously published (11).

The Medical Research Center at Hamad Medical Corporation, Qatar, approved this study and waived the informed consent (Protocol ID: MRC-01-19-135). This study adheres to the RECORD guidelines for reporting observational studies using routinely collected health data (12).

### 2.2 Data Collection

A predefined questionnaire was used for retrospective data extraction from electronic medical records about patient demographics, IVIg dose, clinical indication, number of prescriptions, brand of IVIg, and prescriber specialty. The age of patients was considered at the time they received their first dose of IVIg. The quantity of IVIg per indication was calculated by aggregating all IVIg prescriptions for individual patients under the same clinical indication. The total volume of IVIg consumed was cross-referenced with the HGH drug supply and pharmacy records, which also provided information about the cost of IVIg.

Clinical indications for dispensing IVIg were categorized based on the available evidence from two prominent regulatory authorities: the FDA and the EMA recommendations, and four international guidelines, the American Academy of Asthma Allergy and Immunology (AAAAI, 2016), the Canadian Immunoglobulins Provincial Guidelines and Shortage Framework (Quebec and UK, 2018), the Joint United Kingdom Blood Transfusion and Tissue Transplantation Services Professional Advisory Committee (UK JPAC, 2014) and the National Blood Authority clinical criteria for use of immunoglobulin in Australia (Australian NBA, 2018).

Categories include (1): FDA- and/or EMA-approved indications (2); indications supported by international guidelines where EMA and/or FDA did not approve the indications; (however, at least one international guideline considered it definitely beneficial due to its established treatment role); (3) indications where IVIg has an emerging role as a second-line treatment if first-line medications failed or were not available or contraindicated; (4) indications with little or no supportive evidence or not recommended; (5) indications with ≥ 2 conflicting guideline recommendations; (6) and finally indications not addressed in the reviewed guidelines.

All adverse effects documented during or immediately after the IVIg infusion (within 24 hours), were recorded, and those deemed by the treating physician as associated with IVIg were categorized as IVIg adverse effects. Late-developing complications like acute kidney injury and thrombo-embolic complications were not considered in this analysis unless documented. The documented IVIg adverse effects were further correlated with individual comorbid illness and with IVIg clinical indication looking for any associations. IVIg-related mortality data were retrieved from the hospital records.

Immunoglobulin treatment courses were divided into short-term (for acute medical conditions that required usage for ≤ 6 months duration), and long-term treatment (for chronic medical conditions that required maintenance therapy for > 6 months). Response to treatment was decided based on physician/nurse documentation of either complete improvement and IVIg discontinuation (complete recovery), partial improvement, but still on IVIg (improving), or no improvement and directed towards alternative therapy (no improvement/alternative therapy).

### 2.3 Statistical analysis

All continuous variables were described as mean and standard deviation, while categorical variables were described as numbers and relative frequencies (%). Fractions were rounded to one decimal except for *P*, F and X^2^ values. The percentage of missing values across variables varied between 0 and 24% and the analyses were run only on observations that have a complete data set. The Chi-square (X^2^) and Fisher’s exact probability distribution tests were used for assessing the difference in the distribution of a categorical variable between two or more groups. One-way analysis of variance (ANOVA) was used for exploring interactions between age, clinical indications and response to treatment. For any group comparison, a statistical significance level of < 0.05 was considered significant. All statistical analyses were done using the statistical analysis software SPSS v23.0 (SPSS Inc. USA).

## 3 Results

### 3.1 Demographics

We initially identified 7617 IVIg prescriptions dispensed to 891 patients at HGH over the period of 10 years. After excluding duplicate entries and canceled prescriptions, 7270 IVIg prescriptions, representing 841 patient records, were included in the final analyses. The average number of prescriptions per patient was 8.6 (median 4, IQR 6-2), and the average dose per prescription was 26.7 g.

The age at first dose of IVIg was (mean ± SD) 26.8 ± 21.2 (range 1-95 years). Females represented 46% (384 patients), and 55.2% were ≥ 18 (mean age ± SD) 42 ± 16.2 years. The majority of patients (64.8%) were Arabs, with Qatari patients comprising 313 individuals (37.2%), and 232 (27.6%) being of other Arab descent. Additionally, 259 (30.8%) were non-Arab Asians.

Among the 841 patients, 526 (62.5%) received IVIg in the inpatient general ward, followed by 176 (20.9%) in the intensive care unit, 80 (9.5%) outpatient, and 27 (3.2%) in the emergency room. The three medical specialties most prevalently administering IVIg were neurology (242 patients, 28.8%), hematology (180 patients, 21.4%) and pediatrics (100 patients, 11.9%). A summary of patient characteristics is provided in **Table 1**.

**Table 1:** Characteristics of patients receiving intravenous immunoglobulin treatment from 2009 to 2019 at Hamad General Hospital, Qatar.

Variable	N=841 patients (7270 prescriptions)
**Sex, females, n (%)**	384 (45.7%)
**Age (y)** Mean ± SD, Median (IQR)	26.8 ± 21.2 24.0 (44-6)
Age ≥ 18	464 (55.2%)
Age < 18	377 (44.8%)
Ethnicity n (%) *	
Qatari	313 (37.2%)
Asians	259 (30.8%)
Arab	232 (27.6%)
European	14 (1.7%)
Others	9 (1.1%)
Location of IVIg administration, n (%) †	
General ward	526 (62.5%)
Intensive care	176 (20.9%)
Ambulatory	80 (9.5%)
Emergency department	27 (3.2%)
Specialty of physician prescribing IVIg	
Neurologist	242 (28.8%)
Hematologist	180 (21.4%)
Pediatrics	100 (11.9%)
Nephrologist	55 (6.5%)
Immunologist	52 (6.2%)
Intensivist	40 (4.8%)
Rheumatologist	28 (3.3%)
Other specialty**	47 (6.3%)
Not documented	97 (11.5%)
Amount of IVIg consumed in the 10-yr period (Kg)	193.4
Immunodeficiency, kg (%)	66.1 (34%)
Primary Immunodeficiency diseases	63.4
Immunodeficiency due to secondary causes	1.4
Chronic lymphocytic leukemia	1.21
Neurological disorders	63.6 (33%)
Hematology/oncology disorders	24.0 (12.4%)
Miscellaneous disorders	16.0 (8.2%)
Autoimmune diseases	13.1 (6.8%)
Renal disorders	6.1 (3.2%)
Infectious disorders	4.6 (2.4%)
Comorbid Medical Conditions	
Hypertension	116 (13.8%)
Diabetes	93 (11.1%)
Asthma	45 (5.4%)
Malignancy	37 (4.4%)
Coronary artery disease	17 (2%)
Chronic obstructive pulmonary disease	6 (0.7%)
Rheumatoid arthritis	5 (0.6%)
Other diseases**‡**	264 (31.4%)
No chronic illness	258 (30.7%)

*Out of 827 recorded ethnicities. †Out of 809 available locations **Pulmonologist, cardiologist, dermatologist, Internist, ED physicians. ‡For example, thyroid disease, dyslipidemia, chronic kidney disease and chronic liver disease.

### 3.2 IVIg indications

**Figure 1:**
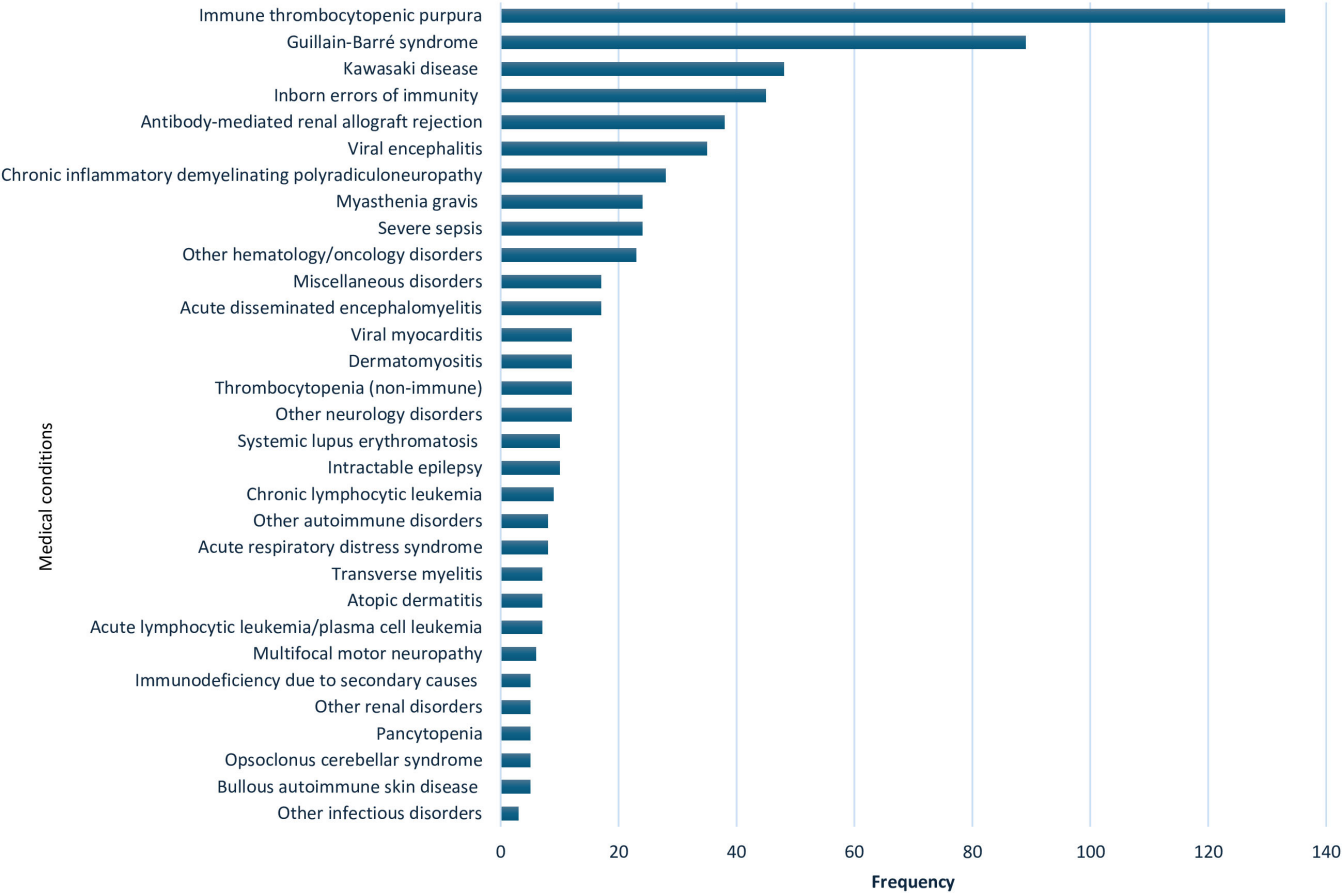
Indications for use of intravenous immunoglobulin in 669 patients from 2009 to 2019 at Hamad General Hospital, Qatar. Other hematology/oncology disorders: Hemolytic disease of the fetus and newborn, neonatal alloimmune thrombocytopenia, parvovirus B19 infection-related chronic pure red cell aplasia, sickle cell crisis, hemophilia (hereditary or acquired), autoimmune hemolytic anemia, Hemophagocytic lymphohistiocytosis, Ewing sarcoma, T-cell lymphoma, febrile neutropenia & Kasabach–Merritt syndrome. Miscellaneous disorders: pulmonary hemorrhage, vasculitis drug eruption, necrotizing enterocolitis, staphylococcal scalded skin syndrome, chronic urticaria, Steven Johnson syndrome & toxic epidermal necrolysis, post lung transplant, dilated cardiomyopathy, and thyroid storm. Other neurology disorders: stiff person syndrome, ataxia, multiple sclerosis, cervical myelitis, acute lumbosacral polyradiculopathy, hereditary sensorimotor neuropathy, autoimmune apraxia, autoimmune paraneoplastic encephalitis, amyotrophic lateral sclerosis and intracranial hemorrhage. Other autoimmune disorders: idiopathic inflammatory polymyositis, systemic juvenile idiopathic arthritis & antiphospholipid antibody syndrome. Other renal disorders: glomerulonephritis and hemolytic uremic syndrome. Other infectious disorders: viral pneumonitis and refractory clostridium difficile infection.

IVIg was used for 63 indications in the 10-year study period. A sum of 172 patients (20%) had no documented indication for IVIg. **Figure 1** summarizes all indications. The top five indications receiving IVIg were: ITP (133 patients; 20%), followed by GBS (89 patients; 13.3%), KD (48 patients; 7.2%), IEI (45 patients; 6.6%), and antibody-mediated renal allograft rejection (ABMR), which accounted for 38 patients (5.7%).

A total of 50 patients received IVIg for primary and secondary immunodeficiencies with a mean age (± SD) 14.4 (± 13.4) years. Following the inborn errors of immunity classification, the most prevalent IEI in this cohort were predominantly antibody deficiencies due to hypogammaglobulinemia (9 patients), common variable immunodeficiency phenotype (8 patients) and X-linked agammaglobulinemia (7 patients). Two patients with IEI required IVIg post hematopoietic stem cell transplantation, while five patients received it for secondary causes of immunodeficiency. Further details are given in **Table 2**.

**Table 2:** Frequency and cumulative dose of intravenous immunoglobulin used for inborn errors of immunity diseases (IEI) from 2009 to 2019 at Hamad General Hospital, Qatar.

Indication	Frequency (n)	IVIg prescriptions		Cumulative IVIg dose (g)
		Mean dose (g)	Mean number	
IEI**, total**	45			63416
Hypogammaglobulinemia	9	29	70.1	19290
Common variable immunodeficiency	8	41	30	6179
X-linked agammaglobulinemia	7	42	79.4	26061
Ataxia-telangiectasia	6	15	53.5	4039
IEI, unspecified	3	8.3	53	1425
Hyper IgE syndrome	2	30	13.5	790
Autoimmune lymphoproliferative syndrome	2	16	55	1232
Specific antibody deficiency (SAD)	2	25	12	570
PIK3CD GOF, chronic EBV infection	1	35	10	350
Thymoma, hypogammaglobulinemia, SAD	1	45	31	1395
LRBA mutation	1	15	39	585
Severe combined immunodeficiency (SCID)	1	10	87	870
SCID, post HSCT	1	15	42	630
IEI, post HSCT	1	–	22	–
**Secondary immunodeficiency, total**	5			1425
Post HSCT for NHL, secondary antibody deficiency	1	35	23	805
Post rituximab, hypogammaglobulinemia	1	30	16	480
Post chemotherapy, secondary immunodeficiency with low IgG and SAD	1	30	2	60
Post HSCT for HL with CMV pneumonitis and GVHD	1	35	2	70
Post HSCT for aplastic anemia	1	10	1	10

N, number of patients; GOF, gain of function; EBV, Epstein-Barr virus; LRBA, lipopolysaccharide responsive beige-like anchor; HSCT, hematopoietic stem cell transplantation; HL, Hodgkin lymphoma, NHL, non-Hodgkin lymphoma; CMV, cytomegalovirus; GVHD, graft versus host disease.

The clinical conditions managed with IVIg in Qatar, along with the corresponding level of scientific evidence based on FDA, EMA, or international guidelines, are illustrated in **Table 3**.

Table 3Clinical conditions treated with intravenous immunoglobulin in Qatar from 2009 to 2019, and their corresponding level of scientific evidence (color coded) based on FDA, EMA, or international guidelines.

**Table:** 

Clinical condition	International recommendations					
	FDA	EMA	AAAAI	Canadian Provincial Criteria	UK JPAC Criteria	Australian NBA Criteria
Inborn Error of Immunity and Secondary Immunodeficiency						
IEI.						
Secondary causes of immunodeficiency.						^1^
Neurologic disorders						
Chronic inflammatory demyelinating polyradiculoneuropathy					^2^	
Multifocal motor neuropathy						
Stiff person syndrome				^3^		
Guillain-Barré syndrome (GBS)						
Myasthenia gravis (MG)						
Viral encephalitis (enteroviral)			^4^	^4^		
Intractable epilepsy				^3^		
Transverse myelitis						
Ataxia						
Multiple sclerosis			^5^	^3^		
Cervical myelitis						
Acute lumbosacral polyradiculopathy						
Hereditary sensorimotor neuropathy						
Autoimmune apraxia						
Autoimmune paraneoplastic encephalitis						
Amyotrophic lateral sclerosis						
Intracranial hemorrhage						
Acute disseminated encephalomyelitis						
Opsoclonus cerebellar syndrome				^3^		
Renal disorders						
Antibody-mediated renal allograft rejection						
Glomerulonephritis						
Atypical hemolytic uremic syndrome						
Hematology/oncology disorders						
Primary immune thrombocytopenic purpura					^6^	
Chronic lymphocytic leukemia						
Post-bone marrow transplantation			^7^	^7^		
Hemolytic disease of the fetus and newborn						
Neonatal alloimmune thrombocytopenia						
Acute lymphocytic leukemia-plasma cell leukemia						
Parvovirus B19 infection-related chronic pure red cell aplasia				^3^		
Sickle cell crisis				^8^		
Thrombocytopenia (non-immune)						
Hemophilia (hereditary or acquired)						
Autoimmune hemolytic anemia						
Pancytopenia						
Hemophagocytic lymphohistiocytosis						
Ewing sarcoma						
T-cell lymphoma						
Febrile neutropenia						
Kasabach–Merritt syndrome						
Autoimmune disorders						
Dermatomyositis						
Idiopathic inflammatory polymyositis						
Systemic juvenile idiopathic arthritis				^3^		
Systemic lupus erythematosus (SLE)					^9^	
Antiphospholipid antibody syndrome			^10^	^3,11^		^12^
Infectious and infection-related disorders						
Kawasaki disease (syndrome)						
Viral myocarditis			^13^			
Severe sepsis			^4^	^14^		
Acute respiratory distress syndrome						
Viral pneumonitis			^15^			
Refractory clostridium difficile infection						
Miscellaneous disorders						
Pulmonary hemorrhage						
Vasculitis drug eruption						
Bullous skin disease (Pemphigus vulgaris, bullous pemphigus & bullous diabeticorum)				^3^		
Necrotizing enterocolitis						
Atopic dermatitis						
Steven Johnson syndrome & toxic epidermal necrolysis				^3^		
Staphylococcal scalded skin syndrome						
Chronic urticaria			^16^			
Post lung transplant, surfactant protein abnormality						
Dilated cardiomyopathy						
Thyroid storm

**Table:** 

Color code	Interpretation	Individual guideline recommendations based on the strength of available evidence			
		AAAAI	Canadian Provincial Criteria	UK JPAC	Australian NBA
	Approved	Definitely beneficial	Recommended, indicated	**Red indications** - conditions for which Ig treatment is considered the highest priority because of a risk to life without treatment	Established therapeutic role
	Approved	Probably beneficial			
	Approved as a second-line treatment	Might provide benefit	Not recommended for routine use but some evidence that IVIg may be considered an option for therapy.	**Blue indications** - conditions for which there is a reasonable evidence base for the use of IVIg, but other treatment options are available	Emerging therapeutic role
	Not approved	Unlikely to be beneficial	Not recommended	**Grey indications** - immune-mediated disorders with limited or little/no evidence	IVIg can be used in exceptional circumstances only
	Not recommended		Contraindicated	Not recommended	Not supported

FDA, Food and Drug Administration (2023); EMA, European Medicine Agency (2021); AAAAI, American Academy of Asthma Allergy and Immunology (2016); Canadian Provincial, Canadian Immunoglobulins Provincial Guidelines and Shortage Framework (Quebec and UK-2018); JPAC, Joint United Kingdom (UK) Blood Transfusion and Tissue Transplantation Services Professional Advisory Committee 2014, NBA, National Blood Authority Clinical Criteria for use of immunoglobulin in Australia 2018; IVIg, intravenous immunoglobulin.

^1^Emerging role in secondary hypogammaglobulinemia not related to hematological malignancies or post hematopoietic stem cell transplantation. ^2^IVIg is optional in long term disease (blue indications). ^3^Disagreement among provincial recommendations. ^4^Only in immunocompromised very severe cases. ^5^Relapsing remitting multiple sclerosis. ^6^Gray indication if chronic immune thrombocytopenic purpura. ^7^Selected patients with chronic graft versus host disease and recurrent serious bacterial infections with a demonstrable defect in antibody production capacity could benefit from IVIg. ^8^If Hemolytic transfusion reaction (hyperhemolysis syndrome). ^9^Without secondary immunocytopenias including juvenile. ^10^In pregnancy. ^11^Catastrophic anti phospholipid antibody syndrome. ^12^Confirmed diagnosis of Catastrophic antiphospholipid syndrome with clinical deterioration. ^13^Acute myocarditis. ^14^Severe neonatal sepsis. ^15^Respiratory syncytial virus pneumonitis (proven for palivizumab). ^16^Delayed pressure urticaria.

A total of 375 patients (56%), received IVIg for FDA- and/or EMA-approved indications (category 1), and 38 patients (6%), were given IVIg for indications approved by one or more international guidelines (category 2). These indications include myasthenia gravis (24 patients), acute lymphocytic leukemia and plasma-cell leukemia (7 patients), polymyositis (PM) (4 patients), hemolytic disease of the fetus and newborn (2 patients), and neonatal alloimmune thrombocytopenia (1 patient) (**Table 4**).

**Table 4:** Frequency and cumulative dose of intravenous immunoglobulin used in Qatar for approved indications during the 10-year period (2009- 2019).

Approved indications (category 1&2)	Frequency (n)	IVIg prescriptions		Cumulative dose of IVIg (g)
		Mean dose (g)	Mean number	
**FDA and/or EMA**	**375 (56%)**			**139800.9 (72.3%)**
Primary immune thrombocytopenic purpura	133	48.1	3.8	18137.9
Guillain-Barré syndrome	89	22.7	9.1	9581.3
Kawasaki disease	48	29	1	1379
Inborn errors of immunity diseases	45	28	51	63416
Chronic inflammatory demyelinating polyradiculoneuropathy	28	41.4	27.4	30957.6
Dermatomyositis	12	34	20	7490.6
Chronic lymphocytic leukemia	9	34.1	5	1216
Multifocal motor neuropathy	6	65.4	33.2	6197.5
Secondary causes of immunodeficiency	5	28	8.8	1425
**Indications approved by other international guidelines**	**38 (5.7%)**			**7510.5 (3.9%)**
Myasthenia gravis	24 (63%)	27.4	8.1	5212.1
Acute lymphocytic leukemia-plasma cell leukemia	7			703
Idiopathic inflammatory polymyositis	4	38	11	1588
Hemolytic disease of the fetus and newborn	2	2	2	7.4
Neonatal alloimmune thrombocytopenia	1	–	–	–

N, number of patients; -, missing data.

Category 3 represented 162 patients (24.3%). The most frequent indications for IVIg usage here as alternative therapy were antibody-mediated renal allograft rejection (ABMR) and viral encephalitis. Most of the 63 indications of IVIg usage in this study fall under this category as it encompasses neurological, hematological, infectious, and dermatological conditions where IVIg was tried due to lack or failure of first-line treatments (**Table 5**).

**Table 5:** Frequency and cumulative dose of intravenous immunoglobulin used in Qatar for indications where it has an emerging role as second-line treatment during the 10-year period (2009- 2019).

Clinical condition (category 3)	Frequency (n)	IVIg prescriptions		Cumulative dose of IVIg (g)
		Mean dose (g)	Mean number	
**Total**	**162 (24.2%)**			**32712.6 (17%)**
Antibody-mediated renal allograft rejection	38	16	6.2	5906.9
Viral encephalitis	35	17.2	4.5	2700
Acute disseminated encephalomyelitis	17	19.4	3.6	1714
Thrombocytopenia (non-immune)	12	34	34	2124.6
Viral myocarditis	12	21	1	249.6
Transverse myelitis	7	21	4.8	835
Atopic dermatitis	7	37.6	3.3	2074
Opsoclonus cerebellar syndrome	5	16.2	19	1417
Bullous Skin disease (Pemphigus vulgaris, bullous pemphigus & bullous diabeticorum)	5	95	27	11545
Parvovirus B19 infection-related chronic pure red cell aplasia	4	43.3	2.3	361
Hemophagocytic lymphohistiocytosis	4	4.1	6.5	82.5
Steven Johnson syndrome & toxic epidermal necrolysis	3	51	6.3	855
Autoimmune hemolytic anemia	3	26.3	35	1635
Sickle cell crisis	2	–	–	130
Hemophilia (hereditary or acquired)	2	25	6	270
Viral pneumonitis	2	–	–	108
Chronic urticaria	1	200	3	615
Refractory clostridium difficile infection	1	20	3	60
Systemic juvenile idiopathic arthritis	1	–	–	30
Stiff person syndrome	1	–	–	–

N, number of patients; -, missing data.

Indications lacking supportive evidence and were not recommended by international guidelines (category 4), accounted for 20 patients (3%), among the cohort’s indications. Out of these, 10 patients received IVIg for intractable childhood epilepsy. Other discouraged indications included: pancytopenia, glomerulonephritis, and amyotrophic lateral sclerosis (**Table 6**).

**Table 6:** Frequency and cumulative dose of intravenous immunoglobulin used in Qatar during the 10-year period (2009- 2019) for indications with conflicting international recommendations, not recommended, or not addressed in the reviewed guidelines.

Indications with conflicting recommendations (category 4)	Frequency (n)	IVIg prescriptions		Cumulative dose of IVIg (g)
		Mean dose (g)	Mean number	
**Total**	**43 (6.4%)**			**8219.8 (4.3%)**
Severe sepsis	24	26	3.8	2024.5
Systemic lupus erythematosus (SLE)	10	25.3	9	2475
Antiphospholipid antibody syndrome	3	31.3	16.6	1550
Multiple sclerosis	2	20.6	29	1032
Atypical hemolytic uremic syndrome	2	23	2	92.3
Autoimmune paraneoplastic encephalitis	1	24.8	39	966
Thyroid storm	1	–	–	80
Indications not recommended (category 5)				
**Total**	**20 (3%)**			**2246.6 (1.2%)**
Intractable epilepsy	10	14.4	7.4	1616
Pancytopenia	5	29	2	306
Glomerulonephritis	3	14.3	4.6	131
Dilated cardiomyopathy	1	8.8	2	17.6
Amyotrophic lateral sclerosis	1	35.2	5	176
Indications not addressed (category 6)				
**Total**	**31 (4.6%)**			**2881.5 (1.5%)**
Acute respiratory distress syndrome	8	37.1	3.7	741
Pulmonary hemorrhage	4	6.5	12.5	349
Necrotizing enterocolitis	3	18.5	1.5	251
Staphylococcal scalded skin syndrome	2	–	–	60
Ewing sarcoma	2	–	–	–
Ataxia	2	5.7	18	225.5
Post lung transplant, surfactant protein abnormality	1	7	13	91
Vasculitis drug eruption	1	–	–	–
Acute lumbosacral polyradiculopathy	1	–	–	–
Kasabach–Merritt syndrome	1	3	1	3
Febrile neutropenia	1	10	1	10
Intracranial hemorrhage	1	–	–	52
T-cell lymphoma	1	30	2	60
Autoimmune apraxia	1	34	22	748
Hereditary sensorimotor neuropathy	1	–	–	–
Cervical myelitis	1	40	5	200

N, number of patients; -, missing data.

A total of 43 patients (6.4%) received IVIg for 7 medical conditions in which the role of IVIg is controversial due to conflicting evidence, with severe sepsis and systemic lupus erythematosus being the most frequent (category 5). Additionally, 31 patients (4.6%) received IVIg for indications not addressed in the reviewed guidelines (category 6) (**Table 6**).

The age at first dose of IVIg varied significantly among different IVIg indications (*P*=0.00; F=12.06). A one-way ANOVA was performed to compare the effect of age on three distinct treatment responses to short and long-term IVIg therapy, which revealed no statistically significant difference in response to short (*P*=0.22; F=1.48) or long-term (*P*=0.11; F=2.23) IVIg treatment in relation to age (**Supplementary Figures S1**, **S2**). Other confounding variables like comorbid illness, treatments other than IVIg and disease severity, may influence the response to IVIg treatment; however, this was not tested.

### 3.3 IVIg adverse effects and safety profile

Out of 841 patients, only 38 (4.5%) had documented side effects, and in 33 patients, adverse effects occurred during or immediately after the IVIg treatment and were documented to be related to IVIg treatment. Headache was the most reported (1.8%), followed by fever and chills (1%). Serious adverse effects, including aseptic meningitis, anaphylaxis requiring adrenaline, and pulmonary edema, were reported in three patients. A further three patients had hyponatremia necessitating hypertonic saline infusion (**Table 7**, **Supplementary Table S1**).

**Table 7:** Adverse reactions reported from 2009 to 2019, during or immediately after intravenous immunoglobulin treatment at Hamad General Hospital, Qatar.

Adverse reactions	N=33/841 patients	Percentage (3.9%)
Headache	15	1.8%
Fever and chills	8	1%
Itching and skin rash	6	0.7%
Abdominal pain, nausea & vomiting	4	0.5%
Tachycardia and/or chest pain	3	0.4%
Hyponatremia	3	0.4%
Dizziness	2	0.2%
SOB, bronchospasm	1	0.1%
Anaphylaxis requiring adrenaline	1	0.1%
Aseptic meningitis	1	0.1%
Pulmonary edema possible TRALI	1	0.1%
> 1 side effect	10	1.2%

TRALI, Transfusion-related acute lung injury.

Different medical comorbidities were recorded in 583 patients (69.3%) (**Table 1**). However, subgroup analyses examining the association between comorbidities and IVIg adverse effects revealed no significant difference in IVIg adverse effects among patients with or without comorbidities, including diabetes (*P*-value 0.92), hypertension (*P*=0.82), asthma (*P*=0.74), and coronary artery disease (*P*=0.3). Patients with IEI had the highest reported adverse effects (11.4%); although the difference in adverse effects was not statistically significant between various IVIg indications (*P*=0.14; X^2^ = 20.9). No IVIg-related mortality was reported during the 10-year study period.

### 3.4 Response to IVIg treatment

**Figure 2:**
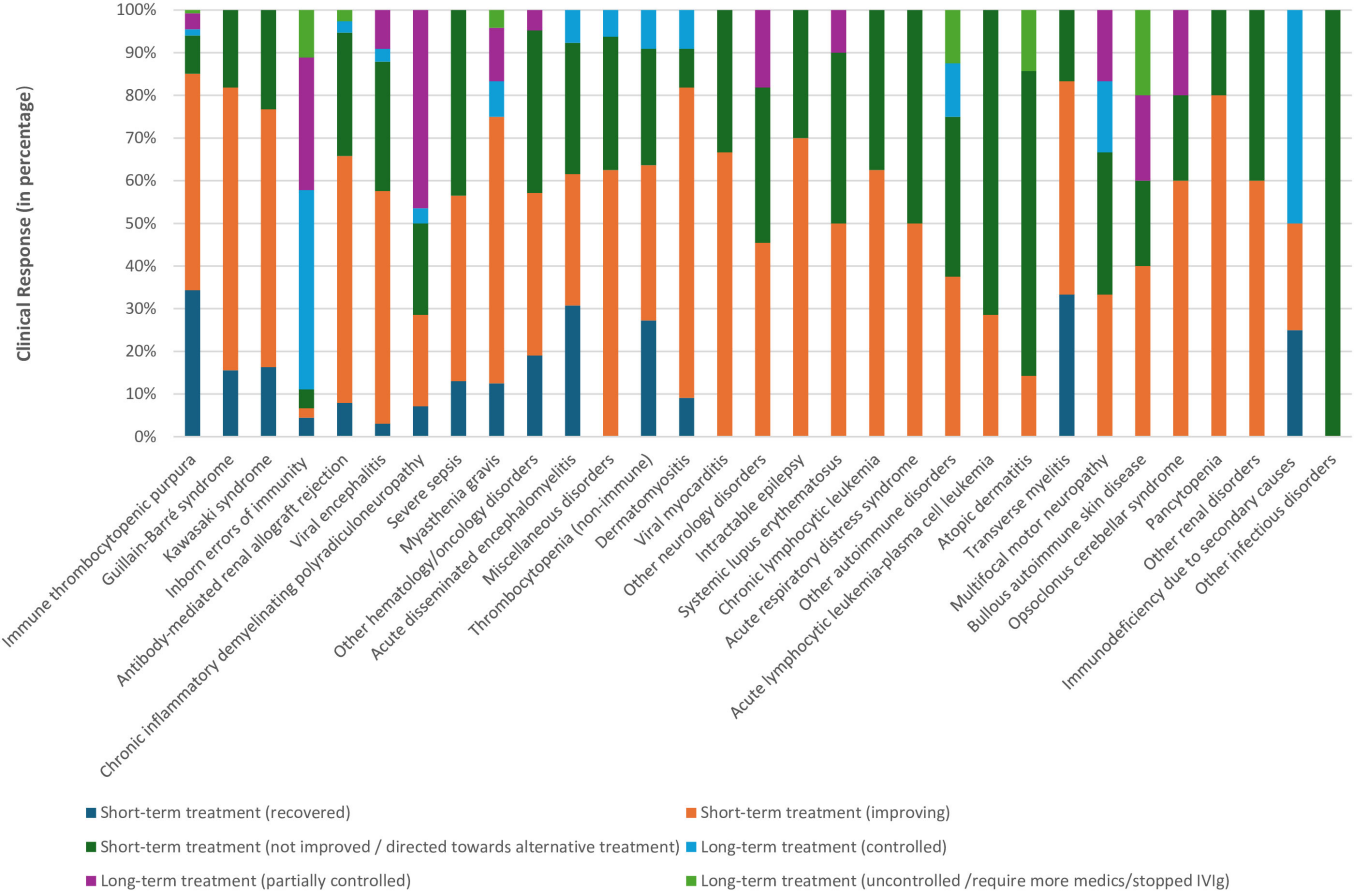
Clinical response to short- and long-term intravenous immunoglobulin treatment from 2009 to 2019 at Hamad General Hospital, Qatar. Other hematology/oncology disorders: Hemolytic disease of the fetus and newborn, neonatal alloimmune thrombocytopenia, parvovirus B19 infection-related chronic pure red cell aplasia, sickle cell crisis, hemophilia (hereditary or acquired), autoimmune hemolytic anemia, Hemophagocytic lymphohistiocytosis, Ewing sarcoma, T-cell lymphoma, febrile neutropenia & Kasabach–Merritt syndrome. Miscellaneous disorders: pulmonary hemorrhage, vasculitis drug eruption, necrotizing enterocolitis, staphylococcal scalded skin syndrome, chronic urticaria, Steven Johnson syndrome & toxic epidermal necrolysis, post lung transplant, dilated cardiomyopathy, and thyroid storm. Other neurology disorders: stiff person syndrome, ataxia, multiple sclerosis, cervical myelitis, acute lumbosacral polyradiculopathy, hereditary sensorimotor neuropathy, autoimmune apraxia, autoimmune paraneoplastic encephalitis, amyotrophic lateral sclerosis and intracranial hemorrhage. Other autoimmune disorders: idiopathic inflammatory polymyositis, systemic juvenile idiopathic arthritis & antiphospholipid antibody syndrome. Other renal disorders: glomerulonephritis and hemolytic uremic syndrome. Other infectious disorders: viral pneumonitis and refractory clostridium difficile infection.

The documented responses to short and long-term IVIg treatment by clinical indication are shown in **Figure 2** and **Supplementary Table S2**. Overall, 545 patients (85.6%) received IVIg for acute illness on a short-term basis, while 92 patients (14.4%) received it as maintenance therapy for chronic medical conditions. Among patients on short-term therapy, 14.8% (94 patients) completely recovered, 48.5% (309 patients) showed improvement, and 22.3% (142 patients) were not improving and directed toward alternative therapy. The highest percentage of recovery was reported in ITP (35%; 46 patients), followed by transverse myelitis (28%; 2 patients). In contrast, more than 80% of patients on long-term treatment were either controlled or partially controlled, with IEI patients, representing 43% of them.

The analyses also showed that clinical responses to IVIg varied significantly based on the treated medical condition (short-term treatment response: *P-*value 0.00; X^2^ = 110.6, long-term response: *P-*value 0.03; X^2^ = 27.39). For example, 86% of ITP patients either recovered, or showed a degree of improvement; while more than two-thirds of acute lymphocytic leukemia and atopic dermatitis patients were not improving and required alternate medication. Similarly, all patients treated with IVIg for viral pneumonitis or refractory clostridium difficile infection failed to demonstrate improvement.

### 3.5 Calculated total dose and projected cost of IVIg

The prescribed dose of IVIg differed among various indications. Overall, IEI patients received 600 – 800 mg/kg replacement doses every 3-4 weeks, while ITP patients were ordered 0.5 g/kg/day, GBS and CIDP 0.4 g/kg/day over 5 days, and KD 1-2 g/kg doses. The average dose and number of prescriptions of IVIg per indication are illustrated in **Tables 2**, **4**–**6**. The estimated total amount of IVIg consumed during the study period was over 190 kg, amounting to an approximate cost of 36 million QAR (approximately 10 million USD). Immunodeficiencies and neurological conditions accounted for the largest quantity of IVIg, with an equal share of 66 kg (34%) and 63.6 kg (33%), respectively. Hematology/oncology diseases received 23.9 kg (12.4%), followed by miscellaneous (15.9 kg; 8.2%), autoimmune (13.1 kg; 6.8%), renal (6.1 kg; 3.2%), and infectious diseases which used 4.6 kg (2.4%) of IVIg.

This cost estimation solely encompasses the price of the vials and does not include overhead costs and expenses related to hospital admissions or daycare provided during administration and monitoring. Neither does it cover the economic estimation of patients’ lost hours and travel time to and from the service.

The usage of IVIg showed a growth trend over the period studied, which is expected. Between 2009 and 2016, 50.5 kg (26%) of IVIg was consumed, with an estimated cost of 7.6 million QAR (approximately 2.1 million USD). In contrast, between 2016 and 2019, 142.9 kg (73.9%) was consumed, costing 28.5 million QAR (approximately 7.8 million USD). (**Supplementary Table S3**). Among the IVIg brands, Privigen^®^ (10 g/100 mL) was the most prescribed, followed by Privigen^®^ (2.5 g/100 mL). Other brands used include KIOVIG^®^ (100 mg/mL) and Intratect^®^ (50 g/L). Of note, in 2020, the cost of Privigen^®^ IVIg increased by around 30%; however, this was not part of the studied time period.

## 4 Discussion

To our knowledge, this is the first exhaustive study evaluating the utilization of IVIg over a 10-year period in Qatar. Our analyses showed that for over 60 indications, increased amounts of IVIg were used over the study period; mostly according to international approval and guideline recommendations. Similarly, with other studies, we identified neurologists, hematologists, and pediatricians as the most frequent prescribers of IVIg in our cohort (13, 14).

In this report, adult patients had a mean age of 42 years when receiving their first IVIg dose. This age is a decade younger than that which is described in similar studies (13, 15). The population of Qatar is distinctively young, which can partly explain the trend towards a younger age group in this sample of patients. Additionally, Qatar has a centralized governmental healthcare system that provides early access to medical care, which may have facilitated the early identification of these patients. There are limited studies on the effect of age on IVIg responsiveness in adults. Burrell et al. (16) showed that younger patients with isolated lower motor neuron syndromes were more likely to respond to IVIg therapy. Our study, however, showed that in adults, responsiveness to IVIg treatment is not age-related.

In this study, we identified 62% of patients receiving IVIg for indications either approved by FDA/EMA or internationally accepted recommendations. In contrast, previous studies described lower rates of between 41% and 45% receiving IVIg for authorized indications (9, 14). A prospective drug utilization study conducted in 13 tertiary Spanish hospitals in 2010 (15), reported a similar utilization rate as shown in our cohort, with 60% of 554 patients receiving IVIg for approved indications. Various factors may contribute to the increased number of approved indications in our research. Firstly, during the last decade, the FDA added three indications for first-line IVIg therapy which are CIDP, MMN and dermatomyositis (DM) (17). As a result, in the previous Spanish study, 86 patients were given IVIg for indications labelled as non-authorized. However, 52 of those patients can be reclassified as their IVIg indications are currently FDA-approved. Furthermore, evidence on the utility of IVIg in other diseases, like GBS and myasthenia gravis, has evolved favoring IVIg over other treatment options (18).

Another point supporting IVIg use is the relative safety and high tolerability of this treatment. This important and beneficial characteristic led to increasing use and authorization of IVIg in special patient groups such as neonates with hemolytic disease of the fetus and newborn, and amongst children in general, who represented over 40% of the population in this study.

In our analysis, a third of the approved IVIg indications were given for treating ITP, and about a quarter for GBS, while around 16% went for IEI disorders and secondary immunodeficiency. Whereas by comparison, the Spanish study (15) reported >70% of approved IVIg usage was for primary and secondary immunodeficiencies. Likewise, a 2004 study from Massachusetts General Hospital found that immunodeficiencies, followed by neurological diseases and ITP, constituted the majority of approved IVIg prescriptions (19). Predominantly antibody deficiency diseases might be underrepresented in our study. Although, a previous pediatric cohort study from Qatar on 131 patients, reported a similarly low rate of IEI diseases and only 23.7% predominantly antibody deficiency requiring IVIg replacement (20). There is significant geographical variation in the prevalence of IEI diseases, with the highest prevalence reported in countries where national IEI databases are available (21). Additionally, demographic and socioeconomic differences, as well as varying levels of IEI awareness among healthcare professionals, may have contributed to the low number of reported IEI cases in this study.

DM received FDA approval in 2020 following a study of 95 patients that demonstrated marked improvement in myositis severity as well as dermatological disease after 40 weeks of high-dose IVIg (22). Other studies showed comparable positive outcomes for polymyositis (PM) when treated with IVIg, with more robust evidence of efficacy in patients who failed to respond to corticosteroids and/or immunosuppressive treatments. IVIg proved especially beneficial for patients with DM/PM who had complications like interstitial lung disease or esophageal involvement and in skin predominant juvenile idiopathic inflammatory myopathy patients (23). In contrast, studies on the response and efficacy of IVIg in inclusion body myositis patients showed fewer promising results (24). While the Australian NBA (25) and the Canadian provincial guidelines (Quebec and UK, 2018) (26), approved IVIg for DM/PM, and inclusion body myositis-associated dysphagia, the NHS England commissioning criteria policy for the use of immunoglobulin of 2021 placed IVIg as fourth-line therapy after failed corticosteroids, rituximab and abatacept biologic therapy (27). This variability underscores the different clinical approaches to using IVIg for inflammatory myopathies across various geographical regions. Our results showed 16 patients (4% of approved indications) received IVIg for DM/PM, but none for inclusion body myositis. Clinical improvement was documented among eight patients with DM, and one patient achieved disease control on long-term IVIg therapy. Despite the small number of patients, these findings support the effectiveness of IVIg therapy in the treatment of these patients.

The Canadian provincial guidelines endorsed IVIg usage as a first-line treatment to prevent and manage acute ABMR, yet Quebec’s Shortage Plans do not prioritize it in the event of an immunoglobulin shortage (26). Similarly, the AAAAI 2016 review supported IVIg use in presensitized patients with features of ABMR due to the encouraging evidence provided (28). However, a recent review of two studies, including one randomized controlled trial and one observational study, on the clinical effectiveness of IVIg in acute ABMR concluded that limited evidence suggested improved renal function in IVIg than methylprednisolone-treated patients (29). Another retrospective study found superior efficacy of high-dose IVIg and bortezomib combination therapy over rituximab-based ABMR therapy (30). Whilst patients with ABMR frequently received IVIg as a second-line indication in this investigation, patients with neurological diseases constituted the majority of recipients. In the Massachusetts General Hospital study (19), ABMR ranked fourth most frequent reason for IVIg use, preceded by IEI, ITP and neurological conditions.

In our study, second-line indications were relatively low and constituted 25% of IVIg indications. This is in comparison with 55% (43 patients in evidence category Ib-IV), in a Malaysian cohort of 78 patients (14), and 31.8% in the Saudi cohort of 305 patients (9). Off-label use of IVIg is a common practice worldwide, especially in elderly, or severely ill patients or when standard treatment is lacking. A good example of this is the use of IVIg during the COVID-19 pandemic, as an adjunctive treatment of SARS-CoV-2 respiratory disease, and to treat vaccine-related thrombotic thrombocytopenia (31). This analysis, capturing data up to March 2019, predates the SARS-CoV-2 pandemic reported in December of the same year, which may therefore account for the relatively low number of off-label IVIg usage.

Additionally, the use of a different categorization method based on the combination of multiple international recommendations helped to effectively categorize some indications based on the strength of available evidence while producing conflicting evidence in others. For example, the AAAAI, UK JPAC, Australian NBA and three out of four of the Canadian Provincial guidelines were concordant on the use of IVIg after failure of systemic corticosteroids and/or immunosuppressive/biologic therapy in autoimmune blistering skin diseases or combination therapy in severe disease form; although, it’s not FDA approved yet. On the other hand, the recommendations were discordant on the role of IVIg in toxic epidermal necrolysis (TEN) and Steven Johnson syndrome (SJS). The AAAAI and UK JPAC prioritized IVIg therapy in these conditions as they have severe and occasionally fatal outcomes, and the usage of high-dose IVIg early on can be lifesaving, in view of limited alternative options. However, the Canadian provincial and Australian NBA guidelines still do not endorse it as upfront therapy due to absence of high-quality evidence. A recent review of 13 systematic review and meta-analysis articles published over the past 10 years, found the use of IVIg and systemic corticosteroids in TEN/SJS remains controversial, and highlighted the potential role of cyclosporine and biologic therapy in the treatment of these conditions (32). Overall, the evidence supporting the use of IVIg in the second-line treatment category is largely observational, but it is rapidly evolving, which makes authorizing and prescribing IVIg in these medical conditions very challenging. Thus, age of the patient, disease severity, quality of life and the availability of effective alternative treatment should be carefully considered when prioritizing IVIg for these patients (33).

In our cohort, IVIg was considered non-beneficial or of unproven effectiveness in only 14% of patients by at least one international recommendation. This emphasizes the good prescribing practices and adherence to international guidelines that are currently in place within Hamad General Hospital. This fair proportion of non-approved use impacted positively on the overall cost as more than 70% of the IVIg cost went for approved indications while non-approved indications collectively consumed 13.3 kg, costing approximately 2.5 million QAR. In fact, the cost of IVIg was not static across the years and is influenced by various factors, including changes in demand and supply dynamics, production costs and currency fluctuations among others. This study was carried out prior to the COVID-19 pandemic; however, there was a 30% increase in the local IVIg cost during the pandemic because of the disrupted supply chains and the significant increase in global demands (34).

Our findings showed that severe sepsis and systemic lupus erythematosus (SLE) were the most frequent indications with conflicting evidence. A systematic review and meta-analysis covering 13 studies (3 controlled trials and 10 observational studies), on the role of therapeutic IVIg in SLE patients found a significant reduction in SLE disease activity scores in the IVIg-treated patients, and a reduction in the dose of systemic corticosteroids by 18 mg; however, the effect of IVIg on complement levels was rather conflicting. The analysis was also limited by the heterogeneity of the clinical manifestations in SLE patients, and the absence of a control group in some studies (35). While the Canadian provincial and AAAAI guidelines supported IVIg use in severe SLE patients unresponsive to or unsuitable for corticosteroids, the Australian NBA and UK JPAC guidelines considered IVIg use unjustifiable, given the availability of alternative therapies such as B cell targeted biological therapy and immunosuppressive medications. In the 10 SLE patients identified in the analyses of our data set, IVIg was prescribed as 3^rd^ or fourth-line treatment in combination with pulse corticosteroids and/or plasmapheresis primarily to treat SLE hematological diseases like thrombocytopenia and microangiopathic hemolytic anemia. This is in-line with the international recommendations against the use of IVIg as 1^st^ line therapy in SLE (36).

There is substantial divergence within the guidelines regarding the role of IVIg in severe sepsis. Early data showed a 6-fold mortality rate reduction in neonates with suspected or proven infection who were given IVIg (37). Yet a recent Cochrane review of 9 studies (3973 infants) found no reduction in mortality or disability during hospital stay up to 2 years of age in the IVIg-treated group compared with placebo or no intervention (38); this was also true for a subgroup given IgM-enriched IVIg.

Despite IVIg generally being well tolerated, with our analyses reporting a 4% rate of immediate adverse effects, the reported adverse reactions in other studies vary from 1% - 81% (39). The retrospective nature of this study and the method of data collection may contribute to the low adverse effects reporting. Moreover, using a limited number of IVIg brands in Hamad General Hospital, and the employment of a standardized infusion protocol may also have helped to minimize adverse effects. In this study it was observed that IVIg was predominantly prescribed in general medical wards rather than emergency department or ICU settings. This distinction is significant because the latter environments are associated with a higher likelihood of inappropriate dosing and off-label indications for IVIg use (40).

This study provides significant initial insight into the real-world applications of IVIg across a large data set, yet it has a number of key challenges and limitations, including the extended duration and the heightened risk of data omissions and record loss, particularly for data sets before the implementation of electronic health records in our institution. Additionally, data on clinical response to IVIg treatment was based on subjective assessments and other factors like comorbid illness, treatments other than IVIg and disease severity may have influenced the reported response.

## 5 Conclusion

This study represents the first in-depth review of IVIg use in Qatar over a decade, showing its broad application in line with global guidelines and a notable use among younger patients for conditions like ITP. Moreover, this study provides valuable information regarding the therapeutic use of IVIg in the treatment of various autoimmune and neurological conditions and emphasizes the possible effectiveness of IVIg in the treatment of ITP and DM/PM, as well as the emerging beneficial role in transverse myelitis. The study also underlines the urgent need for locally established protocols to face the tremendous variability in IVIg prescribing practices and to put in place clear plans to mitigate inevitable shortages of this limited resource, especially given its impact on healthcare costs and the evolving global health landscape.

## Data Availability

The original contributions presented in the study are included in the article/**Supplementary Material**. Further inquiries can be directed to the corresponding authors.

## References

[1] Arumugham VB Rayi A. Intravenous Immunoglobulin (IVIG). Treasure Island (FL: Stat Pearls Publishing (2020). Available at: https://www.ncbi.nlm.nih.gov/books/NBK554446/. 32119333

[2] Immune Globulins. FDA. (2022). Available online at: https://www.fda.gov/vaccines-blood-biologics/approved-blood-products/immune-globulins (accessed July 15, 2023).

[3] European Medicines Agency. Clinical Investigation of Human Normal Immunoglobulin for Intravenous Administration. HS Amsterdam, The Netherlands: European Medicines Agency (2021). Available at: https://www.ema.europa.eu.

[4] Sutton D Visintini S. Off-Label Use of Intravenous Immunoglobulin for Neurological Conditions: A Review of Clinical Effectiveness. Ottawa (ON: Canadian Agency for Drugs and Technologies in Health (2018). Available at: https://www.ncbi.nlm.nih.gov/books/NBK531883/. 30321011

[5] Rajabally YA Uncini A. Outcome and its predictors in guillain–barré Syndrome. J Neurology Neurosurg Psychiatry. (2012) 83:711–8. 10.1136/jnnp-2011-301882 22566597

[6] Clark DE Denby KJ Kaufman LM Fill MMA Piya B Krishnaswami S. Predictors of intravenous immunoglobulin nonresponse and racial disparities in kawasaki disease. Pediatr Infect Dis J. (2018) 37:1227–34. 10.1097/INF.0000000000002019 29570178

[7] Jaime-Pérez JC Aguilar-Calderón P Jiménez-Castillo RA Ramos-Dávila EM Salazar-Cavazos L Gómez-Almaguer D. Treatment outcomes and chronicity predictors for primary immune thrombocytopenia: 10-year data from an academic center. Ann Hematol. (2020) 99:2513–20. 10.1007/s00277-020-04257-2 32945941

[8] Shock A Humphreys DK Nimmerjahn F. Dissecting the mechanism of action of intravenous immunoglobulin in human autoimmune disease: lessons from therapeutic modalities targeting fcγ Receptors. J Allergy Clin Immunol. (2020) 146:492–500. 32721416 10.1016/j.jaci.2020.06.036

[9] Alangari AA Abutaleb MH Albarraq AA Al-Dhowailie AA. Intravenous immunoglobulin utilization in a tertiary care teaching hospital in Saudi Arabia. PubMed. (2008) 29:975–9. 18626524

[10] Burt RK Tappenden P Balabanov R Han X Quigley K Snowden JA. The cost effectiveness of immunoglobulin vs. Hematopoietic stem cell transplantation for CIDP. Front Neurol. (2021) 12:645263. 33828522 10.3389/fneur.2021.645263 PMC8019941

[11] Thalappil S Khalil S Hassini S Al-Nesf M. Subcutaneous immunoglobulin therapy for adult patients with primary immunodeficiency disease: Qatar experience. Qatar Med J. (2023) 2023. 10.5339/qmj.2023.sqac.3 PMC10660831 38025333

[12] Benchimol EI Smeeth L Guttmann A Harron K Moher D Petersen I. The REporting of studies conducted using observational routinely collected health data (RECORD) statement. PloS Med. (2015) 12:e1001885. 26440803 10.1371/journal.pmed.1001885 PMC4595218

[13] Murphy MSQ Tinmouth A Goldman M Chassé M Colas JA Saidenberg E. Trends in IVIG Use at a Tertiary Care Canadian Center and Impact of Provincial Use Mitigation strategies: 10-year Retrospective Study with Interrupted Time Series Analysis. Transfusion. (2019). 10.1111/trf.15271 30916409

[14] Choo SJ Ng CZ Ong YJ Kamarul Baharin KS Chang CT. Intravenous human immunoglobulin utilization patterns and cost analysis in a Malaysian tertiary referral hospital. J Pharm Policy Pract. (2022) 15. 10.1186/s40545-022-00430-2 PMC9040375 35473939

[15] Ruiz-Antorán B Escasany AA Ferraz AV Carreras ID Riba N Escudero SM. Use of non-specific Intravenous Human Immunoglobulins in Spanish hospitals; Need for a Hospital Protocol. Eur J Clin Pharmacol. (2010) 66:633–41. 10.1007/s00228-010-0800-y 20204337

[16] Burrell JR Yiannikas C Rowe D Kiernan MC. Predicting a positive response to intravenous immunoglobulin in isolated lower motor neuron syndromes. PloS One. (2011) 6:e27041. 22066029 10.1371/journal.pone.0027041 PMC3204999

[17] Hooper JA. The history and evolution of immunoglobulin products and their clinical indications. LymphoSign J. (2015) 2:181–94.

[18] Dalakas MC. Update on intravenous immunoglobulin in neurology: modulating neuro-autoimmunity, evolving factors on efficacy and dosing and challenges on stopping chronic IVIg therapy. Neurotherapeutics. (2021) 18:2397–418. 10.1007/s13311-021-01108-4 PMC8585501 34766257

[19] Darabi K Abdel-Wahab O Dzik WH. Current Usage of Intravenous Immune Globulin and the Rationale behind it: the Massachusetts General Hospital Data and a Review of the Literature. Transfusion. (2006) 46:741–53. 10.1111/j.1537-2995.2006.00792.x 16686841

[20] Ehlayel MS Bener A Laban MA. Primary immunodeficiency diseases in children: 15 year experience in a tertiary care medical center in Qatar. J Clin Immunol. (2012) 33:317–24. 10.1007/s10875-012-9812-y 23054346

[21] Weifenbach N Schneckenburger AAC Lötters S. Global distribution of common variable immunodeficiency (CVID) in the light of the UNDP human development index (HDI): a preliminary perspective of a rare disease. J Immunol Res. (2020) 2020:1–8. 10.1155/2020/8416124 PMC7481957 32953893

[22] Aggarwal R Charles-Schoeman C Schessl J Bata-Csörgő Z Dimachkie MM Griger Z. Trial of intravenous immune globulin in dermatomyositis. N Engl J Med. (2022) 387:1264–78. 10.1056/NEJMoa2117912 36198179

[23] Patwardhan A. The value of intravenous immunoglobulin therapy in idiopathic inflammatory myositis in the current transformed era of biologics. Cureus. (2020). 10.7759/cureus.7049 PMC7034746 32128294

[24] Walter MC Lochmüller H Toepfer M Schlotter B Reilich P Schröder M. High-dose Immunoglobulin Therapy in Sporadic Inclusion Body myositis: a double-blind, placebo-controlled Study. J Neurology. (2000) 247:22–8. 10.1007/s004150050005 10701893

[25] Ig Governance. Criteria for the Clinical Use of Immunoglobulin in Australia-3rd Revision (2024). Available online at: https://www.criteria.blood.gov.au (accessed August 10, 2023).

[26] The Canadian national advisory committee on blood and blood products. The National Plan for Management of Shortages of Immunoglobulin Products (Ig). nacblood.ca (2018). Available at: https://nacblood.ca.

[27] NHS England. Commissioning Criteria Policy for the Use of Therapeutic Immunoglobulin (Ig) England (2021). Available online at: https://www.england.nhs.uk/wp-content/uploads/2021/12/cpag-policy-for-therapeutic-immunoglobulin-2021-update.pdf (accessed July 15, 2023).

[28] Perez EE Orange JS Bonilla F Chinen J Chinn IK Dorsey M. Update on the Use of Immunoglobulin in Human disease: A review of Evidence. J Allergy Clin Immunol. (2017) 139:S1–46. 28041678 10.1016/j.jaci.2016.09.023

[29] Khangura SD Visintini S. Off-Label Use of Intravenous Immunoglobulin for Solid Organ Transplant Rejection: a Review of Clinical Effectiveness. Ottawa, ON: Canadian Agency for Drugs and Technologies in Health (2018). Available at: https://www.ncbi.nlm.nih.gov/books/NBK532107/. 30329250

[30] Lachmann N Duerr M Schönemann C Pruß A Budde K Waiser J. Treatment of antibody-mediated renal allograft rejection: improving step by step. J Immunol Res. (2017), 1–9. 10.1155/2017/6872046 PMC5306998 28255562

[31] Tzilas V Manali E Papiris S Bouros D. Intravenous immunoglobulin for the treatment of COVID-19: A promising tool. Respiration. (2020), 1–3. 10.1159/000512727 PMC7801989 33212437

[32] Chang HC Wang TJ Lin MH Chen TJ. A review of the systemic treatment of stevens–johnson syndrome and toxic epidermal necrolysis. Biomedicines. (2022) 10:2105. 36140207 10.3390/biomedicines10092105 PMC9495335

[33] Orange JS Ochs HD Cunningham-Rundles C. Prioritization of evidence-based indications for intravenous immunoglobulin. J Clin Immunol. (2013) 33:1033–6. 10.1007/s10875-013-9912-3 PMC3703306 23764872

[34] Hartmann J Klein HG. Supply and demand for plasma-derived medicinal products – a critical re-assessment amidst the COVID -19 pandemic. Transfusion. (2020). 10.1111/trf.16078 PMC7460929 32856742

[35] Sakthiswary R D’Cruz D. Intravenous immunoglobulin in the therapeutic armamentarium of systemic lupus erythematosus. Medicine. (2014) 93:e86. 25310743 10.1097/MD.0000000000000086 PMC4616295

[36] Fanouriakis A Kostopoulou M Alunno A Aringer M Bajema I Boletis JN. 2019Update of the EULAR recommendations for the management of systemic lupus erythematosus. Ann Rheum Dis. (2019) 78:736–45. 10.1136/annrheumdis-2019-215089 30926722

[37] Jenson HB Pollock BH. The role of intravenous immunoglobulin for the prevention and treatment of neonatal sepsis. Semin Perinatology. (1998) 22:50–63. 10.1016/s0146-0005(98)80007-4 9523399

[38] Ohlsson A Lacy JB. Intravenous immunoglobulin for suspected or proven infection in neonates. Cochrane Database Systematic Rev. (2020). 10.1002/14651858.CD001239.pub6 PMC6988993 31995649

[39] Guo Y Tian X Wang X Xiao Z. Adverse effects of immunoglobulin therapy. Front Immunol. (2018) 9. 10.3389/fimmu.2018.01299 PMC6008653 29951056

[40] Lee JL Saffian SM Makmor-Bakry M Islahudin F Alias H Ali A. Prescribing practices of intravenous immunoglobulin in tertiary care hospitals in Malaysia: a need for a national guideline for immunoglobulin use. Front Pharmacol. (2022) 13. 10.3389/fphar.2022.879287 PMC9218597 35754485

