# Supplementary material for: Intravenous immunoglobulin therapy: usage patterns and response to treatment in Qatar over ten years
Source: Front Immunol. 2024 Dec 2;15:1481079. doi: 10.3389/fimmu.2024.1481079 (PMC11646857; doi:10.3389/fimmu.2024.1481079)

Supplementary Material

1 Supplementary Figures and Tables

1.1 Supplementary Tables

Table S1: Patients with adverse effects occurring during or immediately after intravenous immunoglobulin treatment from 2009 to 2019 at Hamad General Hospital, Qatar.

No	Sex	Indication of IVIg	Type of adverse effect	Brand of IVIg	Dose (g)	Total prescriptions	Intervention
1	F	Polymyositis	Headache	NA	70	5	Analgesia
2	F	ITP	Symptomatic hyponatremia	Privigen 10%	30	2	IVIg withheld & started on hypertonic saline infusion
3	M	ITP	Headache	NA	56	NA	NA
4*	F	PIDD	Bronchospasm, breathlessness, wheeze & fever	NA	NA	135	IVIg withheld. Patient received Diphenhydramine, Hydrocortisone & Salbutamol
5*	F	Polymyositis	Headache, nausea	NA	90	NA	IVIg withheld
6*	F	Myasthenia gravis	Headache, skin rash	NA	40	23	NA
7	M	Antibody-mediated renal allograft rejection	Itching	NA	15	8	NA
8	F	Opsoclonus cerebellar syndrome	Skin rash	NA	NA	5	NA
9	M	Limbic encephalitis	Severe headache	NA	24	6	NA
10	F	Pemphigus vulgaris	Aseptic meningitis	Privigen 10%	112	3	NA
11*	F	Fetal and neonatal alloimmune thrombocytopenia	Chills, tachycardia	NA	43	1	NA
12	F	Guillain-Barre syndrome	Hyponatremia	NA	25	5	NA
13	F	Multifocal motor neuropathy	Hyponatremia	NA	24	43	IVIg withheld & started on hypertonic saline infusion
14*	F	PIDD	Breathlessness, dizziness &back pain	NA	35	15	Slower IVIg infusion rate
15	M	Guillain-Barre syndrome	Headache	NA	26	5	Analgesia

16*	M	ITP	Headache, vomiting	Kiovig 10%	NA	7	Paracetamol, Ranitidine
17	M	ITP	Headache	NA	39	11	Paracetamol
18	F	Antibody-mediated renal allograft rejection	Pulmonary oedema possible TRALI	Privigen 10%	50	7	NA
19	F	Intractable childhood epilepsy	Fever	NA	10	5	NA
20	F	ITP	Headache, fever	Privigen 10%	37	2	Paracetamol, Diphenhydramine
21	M	ITP	Fever	NA	NA	4	NA
22	M	CIDP	Mild fever, headache	Privigen 10%	24	6	NA
23*	F	Juvenile Polymyositis	Chest pain, headache & vomiting	Privigen 10%	40	13	IVIg dose divided into 2 sessions 8-12 hours apart
24	F	ITP	Headache	NA	43	4	Paracetamol, IVIg withheld then resumed at lower rate
25	M	Antibody-mediated renal allograft rejection	Hives & itching	Privigen 10%	40	13	Antihistamine, Hydrocortisone, slower IVIg infusion rate, dose divided into two sessions
26	M	Antibody-mediated renal allograft rejection	Skin rash & itching	NA	40	10	H1 and H2 blockers
27*	F	Kawasaki disease	Chills, tachycardia & whole-body rash	Privigen 10%	35	2	IVIg withheld. Intravenous fluids, Hydrocortisone & Diphenhydramine
28	M	Kawasaki disease	Chills	NA	30	2	Paracetamol & Diphenhydramine
29*	M	Hypogammaglobulinemia – post chemotherapy	Headache, dizziness, & body pain	Privigen 10%	30	36	Intravenous fluids, Hydrocortisone & Diphenhydramine
30	M	Kawasaki disease	Chills & hypotension	NA	30	1	Adrenaline, Diphenhydramine, Hydrocortisone & intravenous fluids. Transferred to Pediatric intensive care unit
31	M	ITP	Headache	Privigen 10%	45	2	NA
32	M	Guillain-Barre syndrome	Headache	Privigen 10%	32	5	NA

* Patients with more than one adverse reaction. IVIg, intravenous immunoglobulin; ITP, immune thrombocytopenic purpura; NA, not available; PIDD, primary immunodeficiency diseases; TRALI, Transfusion-related acute lung injury; CIDP, chronic inflammatory demyelinating polyradiculoneuropathy.

Table S2: Clinical response to short- and long-term intravenous immunoglobulin treatment from 2009 to 2019 at Hamad General Hospital, Qatar.

Indication	Short-term treatment (n=545)			Long-term treatment (n=92)		
	Recovered	Improving	Not improved / directed towards alternative treatment	Controlled	Partially controlled	Uncontrolled /require more medics/stopped IGIV
Total, n (%*)	94 (14.75%)	309 (48.5%)	142 (22.29%)	36 (5.65%)	45 (7.06%)	11 (1.72%)
Immune thrombocytopenic purpura	46 (34.58%)	68 (51.12%)	12 (9.02%)	2 (1.5%)	5 (3.75%)	1 (0.75%)
Guillain-Barré syndrome	12 (13.48%)	51 (57.30%)	14 (15.73%)	0 (0%)	0 (0%)	0 (0%)
Kawasaki syndrome	7 (14.58%)	26 (54.16%)	10 (20.83%)	0 (0%)	0 (0%)	0 (0%)
Primary immunodeficiency diseases	2 (4.44%)	1 (2.22%)	2 (4.44%)	21 (46.66%)	14 (31.1%)	5 (11.1%)
Antibody-mediated renal allograft rejection	3 (7.89%)	22 (57.89%)	11 (28.9%4)	1 (2.63%)	0 (0.00%)	1 (2.63%)
Viral encephalitis	1 (2.85%)	18 (51.42%)	10 (28.57%)	1 (2.58%)	3 (8.57%)	0 (0%)
Chronic inflammatory demyelinating polyradiculoneuropathy	2 (7.14%)	6 (21.42%)	6 (21.42%)	1 (3.57%)	13 (46.42%)	0 (0%)
Severe sepsis	3 (12.5%)	10 (41.66%)	10 (41.66%)	0 (0%)	0 (0%)	0 (0%)
Myasthenia gravis	3 (12.5%)	15 (62.5%)	0 (0%)	2 (8.33%)	3 (8.33%)	1(4.16%)
Other hematology/oncology disorders [1]	4 (17.39%)	8 (34.78%)	8 (34.78%)	0 (0%)	1 (4.34%)	0 (0%)
Acute disseminated encephalomyelitis	4 (23.52%)	4 (23.52%)	4 (23.52%)	1 (5.88%)	0 (0%)	0 (0%)
Miscellaneous disorders [2]	0 (0%)	10 (58.82%)	5 (29.41%)	1 (5.88%)	0 (0%)	0 (0%)
Thrombocytopenia (non-immune)	3 (25%)	4 (33.3%)	3 (25%)	1 (8.33%)	0 (0%)	0 (0%)
Dermatomyositis	1 (8.33%)	8 (66.6%)	1 (8.33%)	1 (8.33%)	0 (0%)	0 (0%)
Viral myocarditis	0 (0%)	8 (66.6%)	4 (33.3%)	0 (0%)	0 (0%)	0 (0%)
Other neurology disorders [3]	0 (0%)	5 (41.66%)	4 (33.3%)	0 (0%)	2 (16.6%)	0 (0%)
Intractable epilepsy	0 (0%)	7 (70%)	3 (30%)	0 (0%)	0 (0%)	0 (0%)
Systemic lupus erythematosus	0 (0%)	5 (50%)	4 (40%)	0 (0%)	1 (10%)	0 (0%)
Chronic lymphocytic leukemia	0 (0%)	5 (55.5%)	3 (33.3%)	0 (0%)	0 (0%)	0 (0%)
Acute respiratory distress syndrome	0 (0%)	4 (50%)	4 (50%)	0 (0%)	0 (0%)	0 (0%)
Other autoimmune disorders [4]	0 (0%)	3 (37.5%)	3 (37.5%)	1 (12.5%)	0 (0%)	1 (12.5%)
Acute lymphocytic leukemia-plasma cell leukemia	0 (0%)	2 (28.57%)	5 (71.42%)	0 (0%)	0 (0%)	0 (0%)
atopic dermatitis	0 (0%)	1 (14.28%)	5 (71.42%)	0 (0%)	0 (0%)	1 (14.28%)
Transverse myelitis	2 (28.57%)	3 (42.85%)	1 (14.28%)	0 (0%)	0 (0%)	0 (0%)

Multifocal motor neuropathy	0 (0%)	2 (33.3%)	2 (33.3%)	1 (16.6%)	1 (16.6%)	0 (0%)
Bullous autoimmune skin disease	0 (0%)	2 (40%)	1 (20%)	0 (0%)	1 (20%)	1 (20%)
Opsoclonus cerebellar syndrome	0 (0%)	3 (60%)	1 (20%)	0 (0%)	1 (20%)	0 (0%)
Pancytopenia	0 (0%)	4 (80%)	1 (20%)	0 (0%)	0 (0%)	0 (0%)
Other renal disorders [5]	0 (0%)	3 (60%)	2 (40%)	0 (0%)	0 (0%)	0 (0%)
Secondary causes of immunodeficiency	1 (20%)	1 (20%)	0 (0%)	2 (20%)	0 (0%)	0 (0%)
Other infectious disorders [6]	0 (0%)	0 (0%)	3 (100%)	0 (0%)	0 (0%)	0 (0%)

*Out of 637 patients.

[1] **Other hematology/oncology disorders**: Hemolytic disease of the fetus and newborn, neonatal alloimmune thrombocytopenia, parvovirus B19 infection-related chronic pure red cell aplasia, sickle cell crisis, hemophilia (hereditary or acquired, autoimmune hemolytic anemia, Hemophagocytic lymphohistiocytosis, Ewing sarcoma, T-cell lymphoma, febrile neutropenia & Kasabach–Merritt syndrome. **Miscellaneous disorders**: pulmonary hemorrhage, vasculitis drug eruption, necrotizing enterocolitis, staphylococcal scalded skin syndrome, chronic urticaria, Steven Johnson syndrome & toxic epidermal necrolysis, post lung transplant, dilated cardiomyopathy, and thyroid storm. **Other neurology disorders**: stiff person syndrome, ataxia, multiple sclerosis, cervical myelitis, acute lumbosacral polyradiculopathy, hereditary sensorimotor neuropathy, autoimmune apraxia, autoimmune paraneoplastic encephalitis, amyotrophic lateral sclerosis and intracranial hemorrhage. **Other autoimmune disorders**: idiopathic inflammatory polymyositis, systemic juvenile idiopathic arthritis & antiphospholipid antibody syndrome. **Other renal disorders**: glomerulonephritis and hemolytic uremic syndrome**. Other infectious disorders**: viral pneumonitis and refractory clostridium difficile infection.

Table S3. Consumption and projected cost of intravenous immunoglobulin during the period 2009–2019 at Hamad General Hospital, Qatar.

ITEM NAME	Unit of measurement	CONSUMPTION (2009-2016)	CONSUMPTION (2016-2019)	Total
**Human normal immunoglobin sucrose free (10-15 gm) intravenous**	Vial	3372	14195	17,567.00
**Human normal immunoglobin sucrose free (2-5 gm) intravenous**	Vial	1686	517	2,203.00
**Projected COST***	QAR	7,579,817.64	28,552,459.65	36,132,277.29
	USD	2,081,652.90	7,841,390.55	9,923,043.45

*** Per vials consumed in each time period.**

1.2 Supplementary figures

Figure S1. The effect of age at first dose of IVIg on short-term therapy outcomes from 2009 to 2019 at Hamad General Hospital, Qatar.

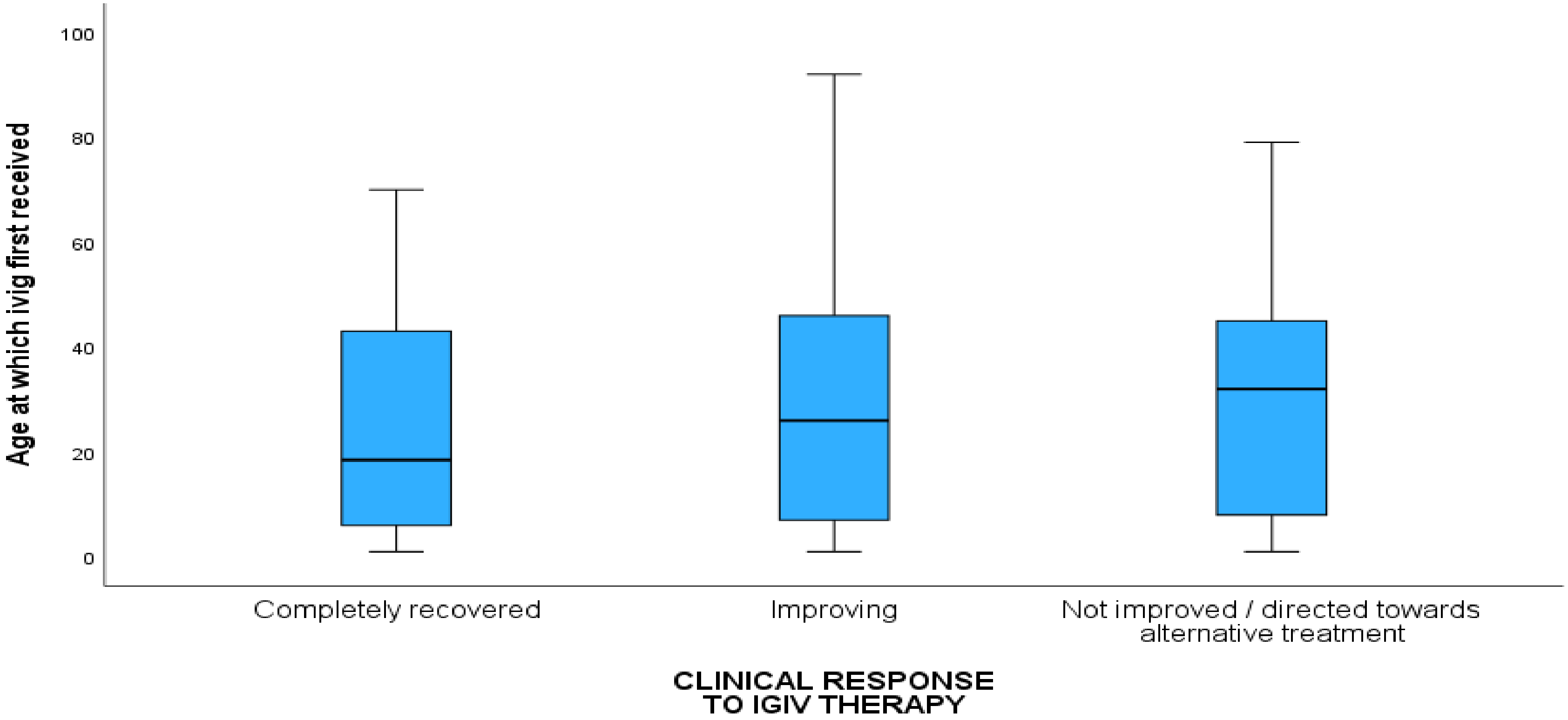

Figure S2 The effect of age at first dose of IVIg on long-term therapy outcomes from 2009 to 2019 at Hamad General Hospital, Qatar.

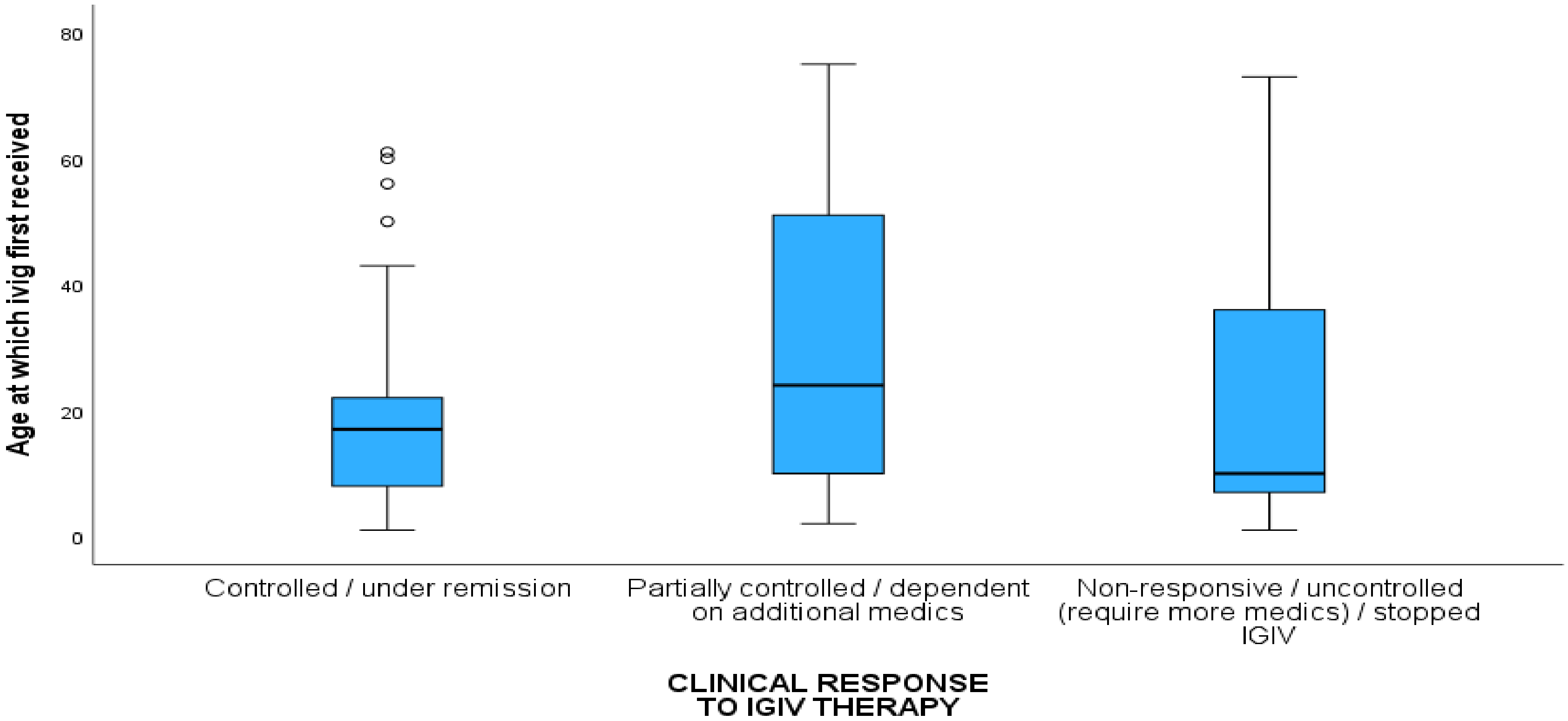

Supplement: Supplementary file 1 [file DataSheet1.pdf]
